# Impfpflichten, Anreize und die effiziente Nutzung von Coronaimpfstoffen

**DOI:** 10.1007/s10273-022-3139-y

**Published:** 2022-03-19

**Authors:** Michael Stolpe

**Affiliations:** Projektbereich Globale Gesundheitsökonomie, Kiel Institut für Weltwirtschaft, Kiellinie 66, 24105 Kiel, Deutschland

**Keywords:** I12, I18

## Abstract

Impfpflichten sind aus ökonomischer Sicht ein negativer Anreiz, sich impfen zu lassen. Im Einzelfall genauso wirksame positive Anreize — etwa staatlich finanzierte Impfprämien — können flexibler gestaltet werden, einige Nachteile starrer gesetzlicher Impfpflichten vermeiden und womöglich eine höhere Impfquote erreichen helfen. Zuverlässige Antikörpertests können helfen, die Subventionierung auf Menschen ohne ausreichenden Immunschutz zu begrenzen. Wegen des hohen volkswirtschaftlichen Werts einer höheren Impfquote im Kampf gegen SARS-CoV-2 hat Deutschland einen großen ungenutzten Finanzierungsspielraum für Impfprämien. Denkbar sind auch Kombinationen von Impfprämien und -pflichten.

## Abstract (en)

From an economic point of view, vaccine mandates are a negative incentive to get vaccinated. Positive incentives that induce unvaccinated individuals just as effectively — such as government-provided cash incentives — can be more flexible and avoid some of the other disadvantages that come with mandates and possibly achieve higher vaccination rates. Given the size of societal benefits from boosting vaccination rates in the fight against SARS-CoV-2, Germany has substantial unused financial leeway to pay for cash incentives. Prudent combinations of cash incentives and vaccine mandates are also worth considering.

Bei den Vorschlägen zu einer Coronaimpfpflicht, die der Deutsche Bundestag aktuell debattiert, geht es eigentlich um finanzielle Impfanreize. Einen physischen Impfzwang oder Gefängnisstrafen fordert niemand. Die Vorschläge zielen vielmehr auf eine bußgeldbewehrte Verpflichtung, innerhalb einer vorgegebenen Frist einen vollständigen Impfschutz nachzuweisen – also auf einen negativen finanziellen Anreiz. Sobald es aber um Anreize geht, stellt sich die Frage ökonomischer Effizienz: Mit welcher Art und Stärke von Anreizen lässt sich eine angestrebte hohe Impfquote in der Bevölkerung am schnellsten erreichen?

Dass Impfanreize überhaupt noch ein Problem sind, wenn hochwirksame Impfstoffe mit für die allermeisten Menschen vernachlässigbar geringem Nebenwirkungsrisiko – wie in Deutschland – frei zugänglich sind, hat mit nicht-monetären Impfkosten zu tun. Nicht-Geimpfte wägen ab zwischen dem erwarteten Nutzen einer Impfung und dem zeitlichen Aufwand einschließlich womöglich unangenehmer kurzfristiger Impfreaktionen, vielleicht vorhandenen Ängsten aufgrund von Informationsdefiziten über Vorteile und Risiken verschiedener Impfstoffe und dem Mut, den es gegebenenfalls braucht, Konformitätsdruck in impfskeptischen Sozialmilieus zu überwinden. Diese Arten von Kosten können verschiedene Menschen das Nutzen-Kosten-Verhältnis einer Impfung unterschiedlich wahrnehmen lassen, selbst wenn der Nutzen für alle gleich wäre.

## Nicht-monetäre Impfkosten

Auch die Impfnutzen können sich unterscheiden. Den höchsten Nutzen haben vulnerable Menschen, die aufgrund von Alter, Vorerkrankungen oder anderen objektiven Risikofaktoren einen schweren Krankheitsverlauf besonders fürchten müssen. Allerdings kann die tatsächliche Schutzwirkung einer Impfung bei Menschen mit geschwächtem Immunsystem geringer ausfallen als bei jenen, in denen die Impfung ein starkes Immunsystem aktiviert. Deren Impfung kann – solange sie die eigene Infektiosität hinreichend verringert (Lyngse et al., 2022a; Mostaghimi et al., 2022) – in ihrem sozialen Umfeld lebende Menschen mit schwachem Immunsystem vor Ansteckung mitschützen, eventuell sogar einen größeren Beitrag zum Schutz dieser Menschen leisten, als sie durch eigene Impfung erreichen können. Zudem sind vulnerable Menschen, auch wegen anderer Krankheitsrisiken als COVID-19, ganz besonders auf den Schutz der knappen Ressourcen des Gesundheitssystems angewiesen, den eine hohe Impfquote bewirken kann.

Die positiven externen Impfeffekte können insbesondere in vulnerablen Menschen eine Zahlungsbereitschaft dafür entstehen lassen, diejenigen zur Impfung zu bewegen, die als Ungeimpfte mit geringem Risiko für einen schweren Verlauf auf Impfschutz wenig Wert legen, aber durch ihre

Impfung zum Schutz der Vulnerablen und des Gesundheitssystems beitragen können. Besonders effektiv könnten solche Zahlungen an junge sozial überdurchschnittlich aktive Menschen sein, die auch ungeimpft oft wenig Anreiz verspüren, soziale Distanz zu praktizieren, und deren Impfung deshalb besonders stark zur Unterbrechung von Infektionsketten beitragen kann. Weil mRNA-Impfstoffe auch bei Omikron meist eine relevante Verringerung der potenziellen Infektiosität erreichen dürften (Lyngse et al., 2022b), kann jede solche Impfung das virale Übertragungsrisiko in zahlreichen Sozialkontakten senken, die diese Menschen oft haben. Gezielte Subventionen, propagiert als Aufwandsentschädigung oder Impfprämie für Impffaule, können dann maßgeblich zu einer effizienten Nutzung der Impfstoffe auf Populationsebene beitragen.

Die Anreizproblematik aufgrund externer Effekte wird überlagert von unvollständiger Information und einer ungleichen Informationsverteilung über das Nutzen-Kosten-Verhältnis einer Impfung in unterschiedlichen sozialen Milieus. Individuelle Informationsnachfrage und Offenheit für angebotene Information sind dabei nicht unabhängig von dem vorab erwarteten Nutzen des Impfschutzes; ist dieser sehr gering, kann womöglich auch kostenlos angebotene Information keine hinreichend große Korrektur der Nutzenerwartung bewirken, um eine Impfnachfrage auszulösen. Eine sehr geringe Nutzenerwartung kann sich – etwa in bildungsfernen Schichten oder Migrantenmilieus – dadurch verfestigen, dass schlecht informierte Ungeimpfte die beobachtete oder vermutete Nicht-Impfung ihrer Peers im eigenen sozialen Umfeld als Beleg für einen geringen Nutzen werten – nach dem Motto: Wäre die Impfung nützlich, hätten sich die anderen doch längst geimpft.

## Impflücken können sich verfestigen

Individuelle Impfanreize und die Informationsnachfrage Ungeimpfter sind keine statischen Phänomene, sondern verändern sich mit zunehmender Impfquote. Genesene sind dabei nicht mehr mit Geimpften gleichzusetzen, weil die Antikörper Genesener nach einer Omikron-Infektion relativ spezifisch für diese Virusvariante zu sein scheinen (Suryawanshi et al., 2022) und schon nach wenigen Monaten oft nicht einmal mehr vor einer erneuten Omikron-Infektion ausreichend schützen. Vollständig Geimpfte und mRNA-Geboosterte dagegen entwickeln Antikörper, die gegen schwere Erkrankungen sowohl durch ältere Corona-Virusvarianten als auch durch Omikron länger Schutz bieten.

Zudem entwickeln nach vollständiger Impfung zum ersten Mal Infizierte ebenso wie erst nach einer Genesung Geimpfte eine Art Superimmunität; Impfung und Infektion ergänzen sich also beim Aufbau von Immunität (Bates et al., 2022). Weiterhin würde ein Laufenlassen des Virus viele vermeidbare Todesfälle zur Folge haben, sodass eine rasche Durchseuchung der gesamten Bevölkerung mit Omikron keine Alternative zu einer hohen Impfquote sein kann.

Je größer der Anteil bereits Geimpfter in einer Population, umso geringer die Wahrscheinlichkeit für noch nicht Geimpfte, sich zu infizieren, und umso schwächer auch ihr Anreiz, sich vorbeugend impfen zu lassen. Dies entspricht dem Präventionsparadoxon: Sind Präventionsmaßnahmen wie das Erreichen einer hohen Impfquote erfolgreich, können sie anschließend als unnötig erscheinen. Diese Fehleinschätzung kann durch kognitive Heuristiken verstärkt werden, die viele Menschen die Wahrscheinlichkeit eines schweren Krankheitsverlaufs unterschätzen und die einer sehr seltenen Impfkomplikation überschätzen lässt. Die Informationsnachfrage noch Ungeimpfter und die Wirksamkeit von Aufklärungskampagnen gehen zurück. Gleichzeitig kann Konformitätsdruck in impfskeptischen Sozialmilieus zunehmen – auch weil die identitätsstiftende Wirkung der Impfablehnung steigen kann, wenn Impfen außerhalb des Milieus zu einer sozialen Norm geworden ist.

Verhaltensökonomische Forschung, wie z. B. Schmelz und Bowles (2021), zeigt: Nicht allein das Virus, sondern auch Impfen ist ansteckend. Dabei ist die Erwartung von Reziprozität zentral. Menschen sind eher zur Impfung bereit, wenn sie erfahren, dass sich dann andere in ihrem Umfeld ebenfalls impfen lassen. Individuelles Vertrauen auf Impfreziprozität scheint zudem positiv mit dem allgemeinen Vertrauensniveau zwischen Menschen korreliert zu sein, das in einer Gesellschaft oder einer sozialen Gruppe über einen langen Zeitraum gewachsen ist – ein Phänomen, das Ökonomen soziales Kapital nennen. Hat ein Land – wie beispielsweise Dänemark – schon vor der Pandemie über Jahrzehnte ein hohes Sozialkapital aufgebaut, kann es in der Pandemie eine hohe Impfquote auch ohne gesetzliche Pflicht und das Setzen finanzieller Anreize rasch erreichen. Länder dagegen, denen das Vertrauen stiftende Sozialkapital fehlt, können es in der Krise nicht herbeizaubern. Diese Länder können es dann ohne Impfpflichten oder gezielte positive Anreize kaum schaffen, eine hohe Impfquote zu erreichen.

## Was hilft besser – negative oder positive Anreize?

Die Regierung mag eine allgemeine Impfpflicht als kostengünstigen Königsweg zu einer hohen Impfquote ansehen, da positive finanzielle Anreize aus Steuern finanziert werden müssten, während Bußgelder für die Nichteinhaltung der Impfpflicht dem Staat zusätzliche Einnahmen bescheren. Ökonomischer Theorie zufolge sollte es für jede Art bußgeldbewehrter Impfpflicht, die einen negativen Anreiz in Höhe der erwarteten Bußgelder für Impfverweigernde impliziert, eine gleich wirksame Impfprämie geben. Dem berühmten Coase-Theorem folgend lässt sich die „Verzerrung“ individueller Impfentscheidungen aufgrund der positiven externen Schutzwirkung des Impfens „unabhängig von der Verteilung der Eigentumsrechte“ korrigieren, wobei es hier um das individuelle Recht auf körperliche Unversehrtheit geht, das Impfprämien respektieren, während Impfpflichten es in Bezug auf das Impfen negieren.

Der Regierung mag eine Impfpflicht auch besser mit dem allgemeinen Gleichheitsgrundsatz vereinbar erscheinen, setzt sie doch eine für alle Menschen gleichermaßen geltende Norm. Wird aber die Höhe der Bußgelder nicht nach dem Einkommen differenziert, kann die Impfpflicht in Konflikt mit dem Ziel sozialer Gerechtigkeit geraten. Wird das Bußgeld daher sozial gestaffelt, kann es andererseits einen Teil seiner abschreckenden Wirkung verlieren, der auf der Aversion vieler Menschen gegenüber finanziellen Verlusten beruht. Verlustaversion beeinflusst menschliche Entscheidungen oft stärker als die Aussicht auf äquivalente finanzielle Gewinne, was gegen Impfprämien sprechen könnte.

Zu den potenziellen Nachteilen einer Impfpflicht gehört allerdings auch die Gefahr, dass allein ihre Einführung die Impfbereitschaft bei manchen Ungeimpften nicht steigen, sondern zurückgehen lassen könnte. Der psychologischen Reaktanztheorie zufolge könnten manche die Pflicht als einen so schwerwiegenden Verlust von Entscheidungsfreiheit empfinden, dass sie ihren Status als Ungeimpfte deshalb höher bewerten und erst recht bewahren wollen. Auch könnte eine Impfpflicht von Menschen in einer liberalen Gesellschaft als Misstrauenserklärung seitens der Regierung gewertet werden und eine bereits vorhandene intrinsische oder altruistische Impfbereitschaft untergraben. Zudem könnten schlecht informierte Impfskeptiker:innen die Pflicht als Signal werten, dass die Impfstoffe nicht überzeugen.

Die Durchsetzung von Impfpflichten kann zudem erhebliche administrative, polizeiliche und juristische Kosten und weitere unerwünschte Nebenwirkungen auslösen. Impfpflichten könnten am Ende nicht nur für Impfverweigernde, sondern auch für die Gesellschaft teurer sein als positive Impfanreize. Schon die aktuell vielfach geäußerte Befürchtung, die bereits vom Deutschen Bundestag beschlossene Impflicht für Beschäftigte in Krankenhäusern und Pflegeheimen ab Mitte März 2022 könnte zu einer Kündigungswelle impfunwilliger Pflegekräfte führen, deutet auf erhebliche Schwierigkeiten und Kosten bei der Umsetzung hin. Der Erfolg einer allgemeinen Impfpflicht könnte durch eine Flut individueller Klagen vor Gericht untergraben werden, zumal es aus medizinischen Gründen von vornherein unvermeidlich erscheint, Ausnahmen zuzulassen.

Beschränkt man zur Minimierung dieser Kosten und Risiken die Impfpflicht auf Alte und andere besonders vulnerable Gruppen, erreicht man jene nicht, deren Impfung aufgrund ihres stärkeren Immunsystems die Vulnerablen und das Gesundheitssystem besonders effektiv mitschützen würde.

## Vorteile von Impfprämien

Ein Großteil der administrativen, polizeilichen und juristischen Probleme von Impfpflichten entfallen, werden stattdessen positive Anreize in Form von Impfprämien gesetzt. Sie lassen sich schneller einführen und umsetzen, haben weniger rechtliche Risiken und stellen möglicherweise ein milderes Mittel im Sinne der Kriterien des Ethikrates dar, das noch nicht ausgereizt ist. Impfprämien könnten zugleich fair und effektiv sein, weil viele, die jetzt in Deutschland noch nicht geimpft sind, einkommensschwachen Gruppen angehören, die erfahrungsgemäß überdurchschnittlich stark auf positive finanzielle Anreize reagieren. Im Einklang mit sozialer Gerechtigkeit würde bei Finanzierung aus progressiven Einkommensteuern die höhere Zahlungsbereitschaft Reicher zur Vermeidung von Mortalitätsrisiken genutzt, um die Impfung ärmerer Menschen zu subventionieren.

Dass Impfprämien tatsächlich einen wirksamen Anreiz setzen können, wird durch neuere empirische Studien belegt. So ließ in einer viel beachteten randomisierten Kontrollstudie aus Schweden, die im Oktober 2021 in Science erschienen ist (Campos-Mercado et al., 2021), schon ein kleiner finanzieller Anreiz in Höhe von 200 Schwedischen Kronen (ca. 20 Euro) die Zahl der Impfungen gegen COVID-19 statistisch signifikant um 4,2 Prozentpunkte steigen. Dagegen hatten die gleichzeitig untersuchten nicht monetären verhaltensökonomischen Interventionen (auch „Nudging“ oder Anstupsen genannt) und die bloße Bereitstellung zusätzlicher Information keinen statistisch signifikanten Einfluss auf Impfentscheidungen. Die positive Wirkung des finanziellen Anreizes war unabhängig vom Alter, Geschlecht und Bildungsgrad der Probanden, aber bei solchen mit niedrigem Sozialstatus höher als bei jenen mit hohem Sozialstatus. Der Grenznutzen des Geldes sinkt bekanntlich mit der Höhe des Einkommens.

Für Deutschland relevant könnten auch die Ergebnisse einer quasi experimentellen Studie zum Blutspenden sein, in der die unerwartete Abschaffung finanzieller Aufwandsentschädigungen in einem süddeutschen Blutspendezentrum zu einem statistisch signifikanten und persistenten Rückgang der Spendenbereitschaft führte (Becker et al., 2019).

Für unterschiedliche Gestaltungsvarianten von Corona-Impfprämien gibt es in Deutschland einen großen finanziellen Spielraum. Der volkswirtschaftliche Wert eines Impfschutzes vor COVID-19 mit 95 % Wirksamkeit wurde in einer jüngst im Quarterly Review of Economics and Finance erschienenen Studie für Deutschland auf 6.341 Euro pro Person geschätzt, was die von der deutschen

Regierung bezahlten Impfstoffpreise für eine vollständige 2-fach-Impfung plus Booster mit mRNA-Impfstoffen um ca. das Hundertfache übersteigt (Gandjour, 2022). Diese Schätzung berücksichtigt allerdings noch nicht die Omikron-Variante des Virus, die den volkswirtschaftlichen Wert von Impfungen mit den existierenden mRNA-Vakzinen gesenkt haben dürfte; deren Wirksamkeit gegen die Fluchtmutation liegt wohl deutlich unter 95 %.

Trotz reduzierter Wirksamkeit und der zudem auch für Ungeimpfte geringeren Wahrscheinlichkeit eines schweren Krankheitsverlaufs nach Omikron-Infektion könnte eine optimal gestaltete Impfprämie auch dann noch vorteilhaft sein, wenn dafür im Einzelfall deutlich mehr gezahlt werden müsste, als in Deutschland üblicherweise für Aufwandsentschädigungen beim Blutspenden oder als Anreiz in der schwedischen randomisierten Kontrollstudie für eine COVID-19-Impfung gezahlt wurde. Dies wird umso mehr gelten, wenn angepasste Impfstoffe mit einer hohen Wirksamkeit gegen Omikron verfügbar werden oder sich neue Virusvarianten mit höherer Letalität durchsetzen. Wie hoch die optimale Impfprämie jeweils wäre, müsste allerdings in weiteren gesundheitsökonomischen Studien erst noch quantifiziert werden.

## Anreizwirkung optimieren

Effizienz und Akzeptanz von Impfprämien ließen sich in der Praxis auch dadurch optimieren, dass man ihre Variabilität über die Zeit und weitere epidemiologisch relevante Dimensionen voll ausschöpft. Die Anpassungsfähigkeit und Individualisierung positiver Anreize eröffnet Chancen auf Effizienzgewinne, die Impfpflichten schon aufgrund starrer rechtlicher Rahmenbedingungen kaum bieten können. Um das Impftempo zu beschleunigen, könnten Impfprämien z. B. zeitlich oder bis zum Erreichen einer aus epidemiologischen Gründen vorgegebenen Impfquote – etwa 95 % aller Erwachsenen – befristet oder aus einem begrenzten, öffentlich bekannt gemachten Budget nach dem Windhundverfahren vergeben werden. Alternativ könnte die Prämienhöhe nach einer vorab festgelegten Formel im Zeitablauf oder abhängig vom Impffortschritt auf Populationsebene fallen.

Um regional konzentrierte Impfskepsis zu adressieren, etwa in Sachsen oder Bayern, könnte eine regionale Differenzierung z. B. in der Höhe der Impfprämien hilfreich sein. Dabei könnte die Bundesregierung durch konditionale Zuschüsse an Länder oder Kommunen einen Anreiz schaffen, dass diese sich ihrerseits an der Finanzierung höherer Impfprämien beteiligen. Bund oder Länder könnten auch einen Wettbewerb zwischen Kommunen oder Landkreisen um die höchste Impfquote in Gang setzen, indem sie attraktive Preise in Form substanzieller Finanztransfers zur Belohnung des größten prozentualen Impffortschritts oder der höchsten erreichten Impfquote innerhalb eines vorgegebenen Zeitraums ausschreiben.

Eine milieuspezifische Differenzierung von Impfprämien könnte Teil einer umfassenderen Strategie gegen milieu- oder gruppenspezifisch verfestigte Impfablehnung werden. Impfprämien könnten dazu gezielte Aufklärungskampagnen und das von der Weltgesundheitsorganisation (WHO) empfohlene „Community Engagement“ verstärken, mit direkter Ansprache impfskeptischer sozialer Gruppen durch lokal vernetzte Multiplikatoren wie z. B. religiöse, zivilgesellschaftliche oder kommunalpolitische Führungspersönlichkeiten – etwa dadurch, dass klug gestaltete Prämien nicht nur den vorab erwarteten Impfnutzen, sondern auch die Informationsnachfrage Ungeimpfter gezielt erhöhen.

Weitere Möglichkeiten zur Differenzierung bieten sich im Hinblick auf vulnerable Gruppen – Alte, Immunsupprimierte und Menschen mit anderen Risikofaktoren –, auf potentielle Superspreader – etwa die Angehörigen von Berufen mit vielen kritischen Sozialkontakten wie Pflegende, Lehrende, Sporttreibende und Musiker:innen, insbesondere Chorsänger:innen – und im Hinblick auf die immunologische Wirksamkeit der Impfstoffe. Während das Gesetz bei Einführung einer Impfpflicht binär festlegen müsste, welche der zugelassenen Vakzine zur Erfüllung der Pflicht anerkannt werden, hätte die Regierung bei Impfprämien mehr Flexibilität und könnte deren Höhe nach Wirksamkeit der Impfstoffe differenzieren und anpassen, sobald neue Fluchtmutationen des Virus die relative Wirksamkeit verschiedener Impfstoffe unerwartet verändern.

## Zuverlässige Antikörpertests nutzen

Um zu vermeiden, dass bereits vollständig Geimpfte und Geboosterte sich allein der Impfprämie wegen als ungeimpft präsentieren und weitere Impfungen verlangen, sollte die Auszahlung von einem quantitativ zuverlässigen negativen Antikörpertest mit hoher Spezifität für SARS-CoV-2 abhängig gemacht werden, der auf ein mögliches Fehlen von Immunschutz hinweist. Laut Andreas Bobrowski, dem Vorsitzenden des Berufsverbands Deutscher Laborärzte (BDL), sei unbestritten, dass bei einem Antikörperwert von weniger als 21,8 BAU pro Milliliter (ml) Blut (BAU = Binding Antibody Units, ein Standardmaß der Weltgesundheitsorganisation) kein verlässlicher Immunschutz mehr gegeben ist; ab einem Wert von 44 BAU/ml verfüge die getestete Person sehr wahrscheinlich über einen Immunschutz (Schneider, 2022).

Zwar ist sich die Wissenschaft noch nicht einig, ob Genesene durch aktive T-Zellen auch ohne nachweisbare Antikörper einen guten Schutz gegen das Virus haben können und ab welchem BAU-Grenzwert ein voller Immunschutz als gegeben gelten kann. Aber darum geht es bei der Feststellung einer Impfprämienberechtigung gar nicht. Es geht stattdessen darum, unter Personen, die behaupten, noch nicht geimpft zu sein, jene zu identifizieren, die möglicherweise keinen ausreichenden oder noch nicht den bestmöglichen erreichbaren Immunschutz haben und von deren Erst- oder Auffrischungsimpfung sie selbst und auch die Gesellschaft profitieren könnten.

Eine empirische Studie des Lübecker Gesundheitsamts und der Universität Lübeck, die am 10. Februar 2022 auf dem Preprint Server „medRxiv" zur Verfügung gestellt wurde (noch ohne Peer Review; Schiffner et al., 2022) schlägt vor, Personen mit einem Antikörperwert von mehr als 200 BAU/ml als ausreichend immungeschützt einzustufen. Ein klar darüber liegender Grenzwert – z. B. 400 oder 500 BAU/ml – erscheint geeignet, die Wahrscheinlichkeit einer irrtümlichen Ablehnung von Personen ohne ausreichenden Immunschutz bei der Prämienauszahlung sehr niedrig zu halten. Die implizite Bevorzugung Immunsupprimierter ist epidemiologisch wünschenswert, die irrtümliche Prämienauszahlung an einige falsch klassifizierte bereits Geimpfte oder Genesene mit ausreichendem Immunschutz harmlos. Zudem können ungeimpft Genesene ihren Immunschutz in aller Regel durch anschließende Impfung deutlich verbessern und eine Superimmunität erreichen (Bates et al., 2022), die auch sozial wünschenswert ist.

Personen, deren Antikörperwert über dem Grenzwert liegt, bekommen keine Prämie, können sich aber weiterhin kostenlos impfen lassen. Daher ist kein erhöhtes Risiko zu erwarten, dass falsch positiv Getestete das Ergebnis nutzen wollen, um eventuell bestehende Coronaauflagen für Menschen ohne eigenen Immunschutz zu umgehen. Die Berechtigung zur Impfprämie könnte zusätzlich von der Bereitschaft abhängig gemacht werden, sich freiwillig in ein nationales Impfregister eintragen zu lassen; damit kann eine wiederholte Beanspruchung der Prämie ausgeschlossen werden.

Gesucht wird eine kluge Mischung von Interventionen, die den größtmöglichen Impferfolg auf schnellstem Wege sicherstellt. Dazu sollte die Politik auch Kombinationen einer Impfpflicht mit positiven Anreizsystemen in Erwägung ziehen. So könnte es zunächst eine Phase zeitlich befristeter Belohnungen für freiwilliges Impfen geben, die dann im Falle des Nichterreichens der angestrebten Impfquote von einer bußgeldbewehrten Impfpflicht abgelöst wird, damit Ungeimpfte nicht auf eine spätere Erhöhung der Impfprämie spekulieren und einfach abwarten.
